# Main Factors Involved in Thyroid Hormone Action

**DOI:** 10.3390/molecules26237337

**Published:** 2021-12-03

**Authors:** Lorena Tedeschi, Cristina Vassalle, Giorgio Iervasi, Laura Sabatino

**Affiliations:** 1 Institute of Clinical Physiology, CNR, 56127 Pisa, Italy; lorena.tedeschi@ifc.cnr.it (L.T.); iervasi@ifc.cnr.it (G.I.); 2 Fondazione CNR-Regione Toscana Gabriele Monasterio, 56127 Pisa, Italy; cristina.vassalle@ftgm.it

**Keywords:** thyroid hormones, thyroid hormone receptor, integrin, genomic, nongenomic

## Abstract

The thyroid hormone receptors are the mediators of a multitude of actions by the thyroid hormones in cells. Most thyroid hormone activities require interaction with nuclear receptors to bind DNA and regulate the expression of target genes. In addition to genomic regulation, thyroid hormones function via activation of specific cytosolic pathways, bypassing interaction with nuclear DNA. In the present work, we reviewed the most recent literature on the characteristics and roles of different factors involved in thyroid hormone function in particular, we discuss the genomic activity of thyroid hormone receptors in the nucleus and the functions of different thyroid hormone receptor isoforms in the cytosol. Furthermore, we describe the integrin αvβ3-mediated thyroid hormone signaling pathway and its rapid nongenomic action in the cell. We furthermore reviewed the thyroid hormone transporters enabling the uptake of thyroid hormones in the cell, and we also include a paragraph on the proteins that mediate thyroid receptors’ shuttling from the nucleus to the cytosol.

## 1. Introduction

Thyroid hormones (THs) are important regulatory molecules in the human body that mediate many metabolic and developmental processes (e.g., increased basal metabolic rate, lipolysis/lipogenesis, adaptive thermogenesis, etc.) [1,2,3,4]. The principal hormone secreted by the thyroid gland and released into the blood stream is thyroxine (T4). The major metabolite, triiodothyronine (T3), is considered to be the biologically active form of THs and is derived from outer ring deiodination of T4 at peripheral tissue sites by specific enzymes called deiodinases [5]. The action of THs requires several milestones, the best known of which is TH binding to specific nuclear receptors and interaction with target DNA. Furthermore, THs may also act through alternative pathways devoid of interaction with nuclear receptors, leading to a more rapid TH action after binding to plasma membrane or cytoplasmic sites. TH action is generally defined as genomic when it directly involves interaction of the hormone with the nuclear receptor, and nongenomic when TH action is not initiated by interaction with specific nuclear receptors. However, the latter distinction has recently been found to be overly simplistic and misleading since it seems to suggest that the nongenomic pathways lack involvement in the regulation of gene expression [6]. Instead, gene modulation may often occur consequently to nongenomic pathway activation. The aim of this review is to discuss the principal factors regulating TH effects in the cell both at the nuclear level, where a direct interaction of thyroid hormone receptors (TRs) and the DNA target occurs, and in the cytosol, where TR isoforms are involved, at least in part, in rapid nongenomic responses. Furthermore, the integrin αvβ3-mediated effects of TH on specific metabolic pathways are also discussed.

## 2. TH Transporters

Having reached their target tissues, THs enter the cells through specific cell membrane transporters [7] (Table 1). Among the so-called monocarboxylate transporter family, MCT8 is considered very specific for THs, showing the highest affinity for T3 and less for T4. MCT8 was found to transport also inactive metabolites such as reverse T3 (rT3) and 3,3′-T2 [8]. It is notable that mutations in the gene for MCT8 (*SLC16A2*) result in impairment of TH passage through the blood–brain barrier and are associated with severe neurological diseases (i.e., Allan–Herndon–Dudley syndrome) [9]. Interestingly, Mct8-deficient mice present only endocrine dysfunctions and do not seem to have the neurological manifestations observed in humans, which may be due to the recruitment of secondary TH transporters [10]. MCT8 is considered the “primary” TH transporter since it is specific for THs, whereas the “secondary” transporters also mediate the uptake of compounds other than THs, such as MCT10, which belongs to the same family as MCT8 and is also an aromatic amino acid transporter. Other secondary transporters include the organic anion transporter polypeptides (such as OATP1C1, OATP1A2, OATP1A4), which mediate the transport of bile acids and steroid hormones, and the large neutral amino acid transporters (such as LAT1 and LAT2), which mediate the entrance of phenylalanine, tyrosine, leucine, arginine, and tryptophan into cells [7]. 

## 3. Thyroid Hormone Receptors and Nuclear Functions

### 3.1. General Characteristics of Thyroid Hormone Receptors

The biological activities of T3 via transcriptional regulation are mediated by thyroid hormone receptors (TRs) which are encoded by two different genes, called *THRA* and *THRB*, and in humans are located on chromosomes 17 and 3, respectively [11]. The two genes generate different variants via alternative splicing: *THRA*’s main products are TRα1, TRα2, and TRα3, whereas *THRB*’s main products are TRβ1, TRβ2, and, described only in rats, TRβ3 (Table 1).

**Table 1:** Main factors mediating TH action.

	Cellular Localization	Ligand	Factor	DNA Interaction
Nuclear receptors	Nucleus	T3	TRa, TRb	TREs
Extra-nuclear receptors	Cytoplasm/Mitochondria	T3	TRa1 (p46)	mit-TREs
			p43/p33/p30/p38	
Integrin	Cell membrane	T4/T3	Integrin avb3	Absent

T3: triiodothyronine; T4: thyroxine; rT3: reverse T3; MCT: Monocarboxylate transporter family; TR: thyroid hormone receptor; TREs: thyroid response elements; mit-TREs: mitochondrial TREs.

**Figure 1:**
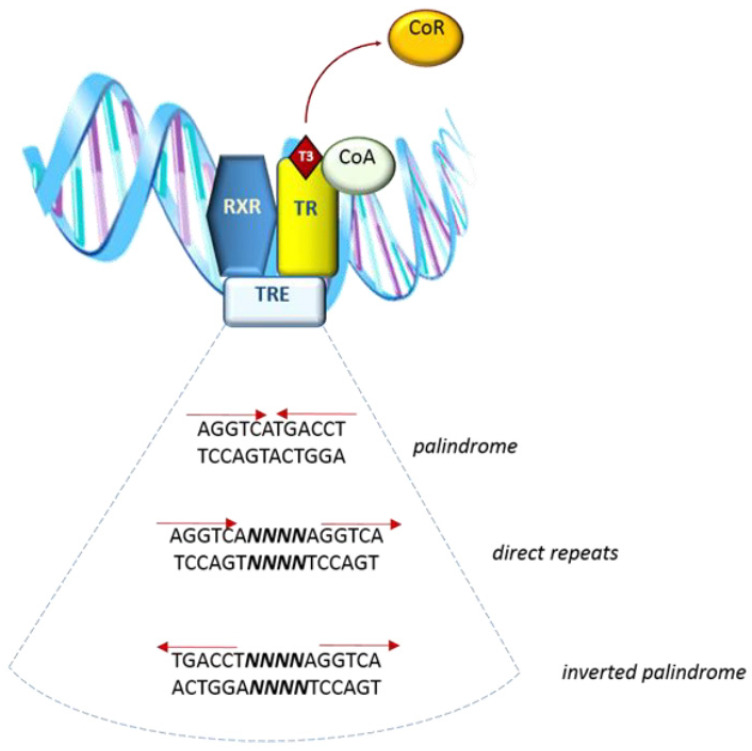
Simplified representation of interaction of the complex T3–TR dimer-CoActivator and TRE sequence in the target gene.

The *THRA* locus contains 10 exons and the spicing site for the TRα2 variant is in exon 9. The *THRB* locus contains 11 exons, among which only exons 3 to 8 are in common to all the TRβ isoforms and present a high homology with TRα isoforms [12]. Direct interaction of T3 with target genes is mediated by nuclear thyroid hormone receptors (TRs), which bind to specific sequences called thyroid response elements (TREs). TREs are located in the regulatory regions of target genes, acting as enhancers (positive TREs) or silencers (negative TREs) of transcription [13] (Figure 1). TREs are formed either by a palindrome hexamer (AGGTCA) called “half-site” or by direct repeats of this sequence, spaced by four nucleotides [14]. TRs belong to a family of nuclear receptors which bind DNA as dimers—more specifically, as homodimers or heterodimers with 9-cis-retinoic acid receptors (RXR)s [15], liver X receptor [16], and PPARs [17]. 

A peculiar aspect of TR is that its regulatory action on positive TREs as a repressor does not require binding to the ligand. Instead, the presence of co-factors (specifically called co-repressors) is needed to silent the target gene. Upon T3 interaction with TR, the co-repressor is released and the following conformational change allows TR to interact with a co-activator, allowing target gene transcription [18] (Figure 1).

### 3.2. TRs’ Multidomain Structure

**Figure 2:**
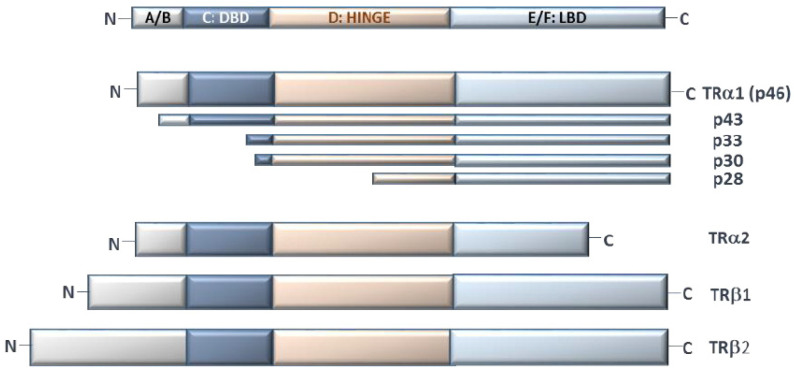
Schematic representation of the main TH isoforms. A/B: N-terminal domain, C: DNA binding domain (DBD), D: hinge region, E/F: ligand binding domain (LBD).

All TRs have a multidomain structure: (1) the A/B domain includes the variable N-terminal region, which is not present in the truncated forms of TRs and is believed to be involved in the interaction with co-factors; (2) the C domain (DNA Binding Domain; DBD) with two zinc fingers, containing specific motifs to recognize DNA (“P-box”) and to distinguish the spacing between half-sites in TREs (“D-box”); (3) the linker D domain, which contains an unfolded hinge to facilitate the rotation between the ligand binding domain (LBD) and DBD; and (4) the E/F domain, with LBD, which displays the interface for homo- or hetero-dimerization. In particular, the E/F domain drives all the conformational changes after ligand binding and interaction with co-regulators. In detail, in the inactive form, LBD binds the co-repressors, which maintain target gene silence; upon T3 binding, helix 12 (in the final part of the protein) relocates to a different position, releasing the co-repressor and displaying a new surface for co-activator recruitment. Co-activators mediate chromatin remodeling (i.e., by histone acetylation or methylation) and the interactions among RNA polymerase and other transcriptional factors [19]. Finally, LBD contains a hydrophobic surface (with heptad repeats) for dimerization [20,21]. TRα2 and TRα3 have an incomplete LBD domain and, although the exact roles of these isoforms are not completely clear, since their dimerization with the full-length receptors is still possible, they probably function only as constitutive repressors of TR-mediated transcription [11,22] (Figure 2).

### 3.3. TR Tissue Distribution

TR isoforms found in human, rat, and mouse tissues have highly homologous amino acid sequences [18]. This conservation among species depends on the fact that there are important specialized functions for each isoform in different organs, and TR isoforms are selectively distributed on the basis of their roles and developmental stage [23]. 

TRα1, TRα2, and TRβ1 are expressed at different relative levels in different tissues, even though TRα1 and TRα2 receptors are mainly present in the central nervous system and muscle, and TRΔα isoforms in the intestine; TRβ1 is the principal isoform in the liver, TRβ2 in the hypothalamus, pituitary, and retina, and TRβ3 and TRΔβ3 are limited to the liver, spleen, heart, and lungs [24,25,26,27]. It is noteworthy that in the liver, all the isoforms α and β show a zonal distribution, suggesting that there might be different sets of genes more specifically activated by one or more isoforms, depending on their location in the organ [28].

Interestingly, there is only a single amino acid difference between the LBDs of the TRα and TRβ receptors, which is a very important issue in the development of TR-isoform-selective analogues for therapeutic purposes [29,30]

Moreover, it has been observed that in several tissues, a critical TR mass, rather than the presence of a particular variant, may be relevant for TH action. Nevertheless, in other conditions, the type of TR isoform may be a more specific determinant. Thus, the fine balancing of the total TR mass and relative composition of TR types, according to the needs of the cell, may explain, at least in part, the diverse range of TH actions in different tissues [31].

### 3.4. TR Nucleus–Cytoplasm Shuttling

The nuclear envelope acts like a selectively permeable barrier. Regardless of direction, the passage requires the presence of the nuclear pore complex (NPC), which acts as regulatory gatekeeper [32]. Nuclear import of small molecules (less than 40 kDa) can occur by passive diffusion through the central channel of the NPCs, whereas import of larger proteins requires an energy-dependent process mediated by specific signal motifs called nuclear localization signals (NLS). The main TR actions occur within the nucleus via interaction with specific target genes responsive to THs. However, TRs can shuttle back to the cytoplasm, crossing the nuclear envelope and being involved in several cellular processes. More specifically, in vitro studies of cell models have shown that the nuclear import of TRα1 involves soluble factors in the cytosol called importin 7, importin β1, and adapter importin α1. This active transport is abolished by exposure to low temperatures [33]. Importins bind TRs at lysine- and arginine-rich nuclear localization signal (NLS1 and NLS2) motifs, localized in the hinge and A/B domains respectively [25]. It is noteworthy that TRβ1, which has no NLS-2 motif, is more abundant than TRα1 in the cytosol. TR transfer from nucleus to cytoplasm is mediated by several exportins and nuclear export signal (NES) motifs in the LBD domain. Exportins include CRM1 (chromosome maintenance factor 1, also known as exportin 1), which cooperates with calreticulin, and exportins 4, 5, 6, and 7 [34]. In vitro studies have shown that overexpression of exportins 5 and 7 leads to a major distribution of TRs in the cytosolic compartment. However, when TRβ1 and exportins 4, 5, or 7 were co-expressed, no variation in T3-mediated gene expression was observed. In contrast, when the unliganded TRα1 was co-expressed with exportin 7, it was less efficient in repressing target gene transcription [34].

The balance between nuclear import and export of TRs is considered a crucial checkpoint for regulation of TH-dependent gene expression, and it is believed that post-translational modifications at several amino acid side chains such as cysteine, serine, threonine, tyrosine, and, above all, lysine (targeted by acetylation, ubiquitylation, sumoylation, methylation) may contribute to the efficacy of these processes [35]. In particular, TRα is a target for acetylation by cAMP-response element-binding acetyltransferase (CBP). This post-translational modification, which occurs at the DBD domain (in Lys residues: K128/K132/K134), ameliorates efficiency of receptor binding to DNA; in fact, substitution of Lys with Arg residues decreases DNA-binding strength and seriously interferes with T3-dependent activation of target genes [36].

### 3.5. TR Extranuclear Functions 

Beside the well-studied functions at the nuclear level, a few emerging roles of TRs in the cytoplasm compartment have been investigated. For example, in normal conditions, several truncated isoforms of TRα have been described at the inner surfaces of the plasma membrane and mitochondria, suggesting their involvement in functions outside the cell nucleus [37]. In particular, the p28 truncated TRα1 isoform, with N-terminal deletion of A/B domain, is located at the mitochondrial inner membrane. Even though the role of p28 has yet to be completely clarified, given its co-localization with adenine nucleotide translocase (ANT) and uncoupling proteins (UCPs), it has been proposed to be involved in the early mitochondrial response to T3 [38]. Another known TRα1 mitochondrial isoform is p43, which binds to T3 with a similar affinity with respect to TRα1 and acts as a potent T3-dependent transcription factor of the mitochondrial genome, mediating the interaction with specific mit-TREs and thus regulating mitochondrial gene expression [39] (Table 1, Figure 2).

## 4. Integrin αvβ3 Mediation of TH Action

**Figure 3:**
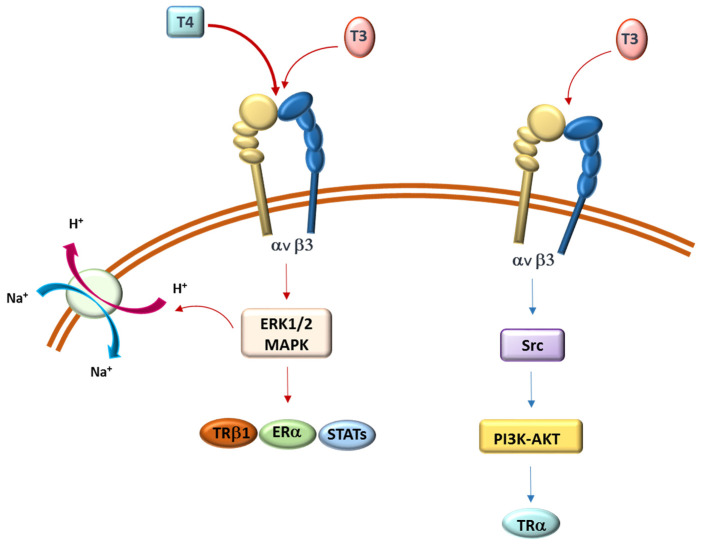
Representation of plasma membrane integrin αvβ3, its interaction with THs (T3 and T4), main cell signaling activated MAPK (ERK1/2) and PI3K–AKT, and main target proteins identified to date (TRβ1, ERα, STATs, Na^+^/H^+^ antiporter, and TRα1) [41,42,43,44,45].

In several conditions, TH effects can be observed within minutes, a very short period for a response requiring transcriptional regulation and, in most cases, T4 is more effective than T3. These signals, independent of ligand binding to nuclear TRs, are mediated at the plasma membrane by protein integrin αvβ3 (Table 1). Integrin αvβ3 is a member of the integrin family, which consists of cell adhesion receptors that play important roles during developmental and pathological processes. The integrin family includes 24 αβ heterodimeric members mediating the attachment of cells to the extracellular matrix (ECM) and involved in specialized cell–cell interactions. Integrin αvβ3 is one of a subset of integrins (8 out of 24); it recognizes the RGD (Arg–Gly–Asp) sequence in the native ligands and has been shown to have a panel of small molecule ligands [40]. Integrin αvβ3 contains a TH-binding domain which binds T4 and, with lower affinity, T3, thus activating PI3K/AKT and MAPK (ERK1/2) downstream pathways [41] (Figure 3). 

At physiological concentrations, T4 is the main ligand for integrin αvβ3 and data obtained with radiolabeled T4 experiments showed that tetraiodothyroacetic acid (tetrac), integrin αvβ3 antibodies, and integrin RGD recognition-site peptide [46] could displace this binding. A study by Cao et al. on CV-1 cells showed that the translocation activity of TRβ1 from the cytoplasm to the nucleus is initiated by T4 at the plasma membrane through the mediation of integrin αvβ3 [42]. In fact, CV-1 cells, which lack nuclear TRs but express plasma membrane integrin αvβ3, once treated with physiological concentrations of T4, show an activation of MAPK pathway which is inhibited by the silencing of integrin αvβ3 [41].

Furthermore, the finding that the occlusion by an antagonist of RGD site of integrin αvβ3 leads to blockage of T4 activity suggests that T4-integrin αvβ3 interaction occurs at or near the RGD site [47]. Molecular modeling studies have demonstrated that the THs and their analogues occupy a much smaller conformational space than does the RGD cyclic peptide (which is the model ligand for RGD binding integrin investigation), suggesting that there can be different possible orientations of THs and analogues in the binding site at the interface between the αv and β3 domains of integrin αvβ3. For the T4Ac analogue, an interaction with both the subunits is the most probable, with the hydroxylated ring toward αv, while T4 molecule is hypothesized to interact with β3 subunit. Other small aromatic ligands interacting with αvβ3 integrin (e.g., steroids and resveratrol) can fit into an alternative binding pocket near the RGD binding site, but this site is too small for the THs because of the bulky iodine [48].

Integrin αvβ3 is highly expressed in activated endothelial cells and newborn vessels, as well as some different cancer cell lines and in primary tissue samples from patients [49]; it also correlates with disease prognosis in various cancer types [50,51,52,53].

After the first evidence of MAPK pathway activation by TH, TH-mediated cell proliferation and cancer cell growth stimulation was demonstrated in several cell lines. The effect on glioblastoma C6, F98, and GL261 cells was measured by accumulation of proliferating cell nuclear antigen (PCNA) and radiolabeled thymidine incorporation. This effect was inhibited by the T4 analogue, tetrac, and by an integrin αvβ3 RGD recognition-site peptide, and it was demonstrated that T3 and T4 are equipotent stimulators of PCNA accumulation in glioblastoma cells. However, the in vivo effect on tumor growth is mainly due to T4, since physiological concentrations of T3 are 50-fold lower with respect to T4 [54].

Through molecular modeling and displacement studies, the existence of two sites capable of T3 binding in human glioma U-87 MG cells was suggested, only one of which also binds T4. Both T3 and T4 activate the MAPK–ERK1/2 pathway and cause cell proliferation, whereas only T3 is also capable of enhancing Src kinase and PI3K activities [43]. Furthermore, TH-activated ERK1/2 has been demonstrated to stimulate estrogen receptor-α (ERα), signal transducer and activator of transcription-3 (STAT3), p53, several TR-associated factors, and sodium-proton exchanger (N^+^/H^+^ antiporter) [44,45] (Figure 3).

By using fluorescently labeled hormones, a selective binding to integrin αvβ3-positive ovarian cancer cells (but not integrin-negative cells) was demonstrated and it was also evidenced that T3 and T4 nongenomically regulate the transcription of integrin monomers in association with basal integrin levels in the cells [55]. This transcriptional regulation occurs in concert with the clustering of the dimeric αvβ3 protein on the cell surface following TH treatment. Similar clustering was shown also in myeloma [56].

The effects of THs when αvβ3 is pathologically overexpressed were evaluated [57] and tetrac or its modified forms (tetrac analogues, nanoparticles formulations) were used for the pharmacological blockade of T4 action at αvβ3 integrin on different cell types [58,59,60,61,62,63]. 

## 5. Conclusions

A multitude of studies have shown that the effects of THs involve both genomic processes and a variety of metabolic pathways. On this basis, the canonical genomic/nongenomic distinction of TH actions must be considered too simplistic and not fully explanatory of all the complex interrelated effects within target cells. On this basis, a better understanding of the main events involving THs in the nucleus, where a direct interaction of TH–TR complex with target DNA occurs, and in the cytosol, where TH regulation requires the activation of specific metabolic pathways (in part involving TR isoforms), may help to create a clearer picture of TH dynamics in target cells.
